# Aplasia Cutis Congenita Scalp Presenting with Life Threatening Hemorrhage: A Case Report

**Published:** 2012-01-01

**Authors:** Farrukh Mahmood, Shahzadi Tasneem, Malik Muhammad Nadeem

**Affiliations:** Department of Pediatric Plastic Surgery, The Children’s Hospital and the Institute of Child Health Lahore, Pakistan; 1 Department of Pediatric Neurosurgery, The Children’s Hospital and the Institute of Child Health Lahore, Pakistan

**Keywords:** Aplasia cutis congenita, Hemorrhage, Superior sagittal sinus

## Abstract

Aplasia cutis congenita is a rare congenital anomaly characterized by the absence of a patch of skin since birth. It may lead to life threatening complications at times. A 5-day-old neonate with Aplasia cutis congenita was received in a state of shock due to tremendous blood loss from the superior sagittal sinus. The neonate was resuscitated immediately followed by closure of the superior sagittal sinus and flap coverage to the defect as a life saving procedure.

## INTRODUCTION

Aplasia Cutis Congenita (ACC) is first described by Cordon in 1767 and characterized by the absence of a patch of skin, usually over the vertex, since birth. In more than 70% of cases it is located on the scalp; however, it may be found over the trunk, face, and extremities. Quite often limited to dermis or epidermis but in few cases it may extend to the brain. In most instances, these are managed non-operatively but in few cases where meninges and dural sinuses are involved urgent surgical intervention becomes necessary to avoid hemorrhage and infections [1-4]. Herein a case of ACC presenting with life threatening hemorrhage is being reported; the patient was immediately intervened and his life was saved.

## CASE REPORT

A 5-day-old male neonate was received in the nursery emergency of our hospital in a state of shock. The short history depicted that the neonate was a product of consanguineous marriage and was born in a private hospital through spontaneous vaginal delivery. The neonate had an ulcer over the vertex since birth. The ulcer was being managed with dressings. The neonate was going well when during change of dressing massive hemorrhage started. The medical staff of that hospital tightly packed the wound and referred the patient to our hospital for further management. On examination the patient had severe pallor, cold extremities, and impalpable pulses. Fortunately the blood was arranged in the meanwhile; the patient was transfused and then taken directly to the operation theatre. At operation the defect was identified on the vertex as ACC involving scalp, part of skull bone and meninges. It was found that the ulcer had eroded the superior sagittal sinus thus causing tremendous hemorrhage. The superior sagittal sinus was repaired by the neurosurgeon which resulted in hemostasis. The defect was covered with a transposition flap and donor area was covered with split thickness skin graft (Fig. 1-5). Postoperative recovery was uneventful. The patient was started orally on the same day of the operation. At follow up both the flap and graft were healthy; the thigh wound was also healing. The patient is doing well now.

**Figure:**
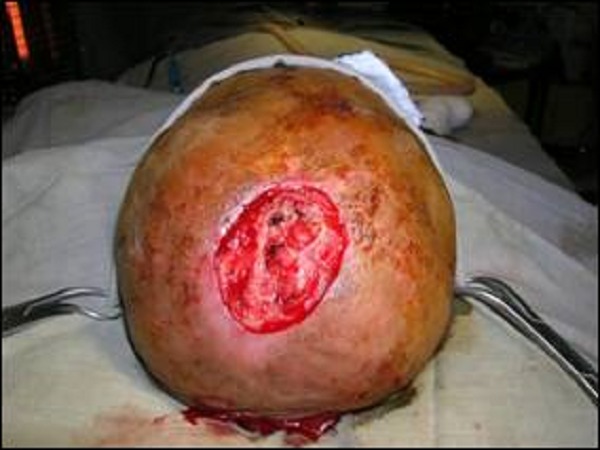
Figure 1: Wound Debridement and repair of superior sagittal sinus

**Figure:**
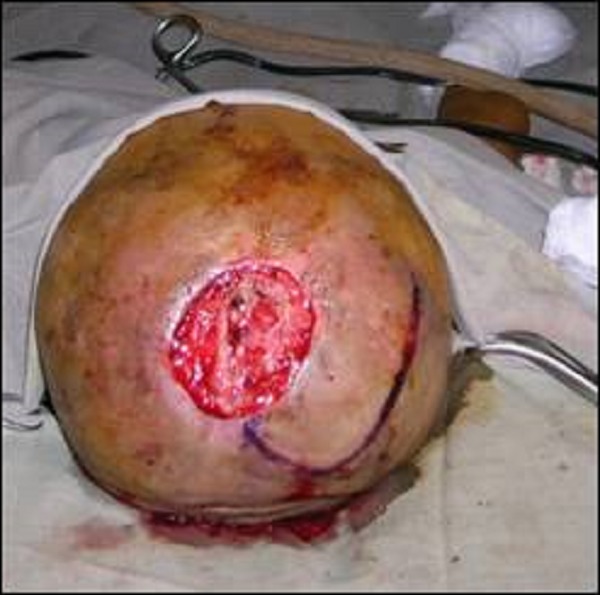
Figure 2: Transposition Flap Marking

**Figure:**
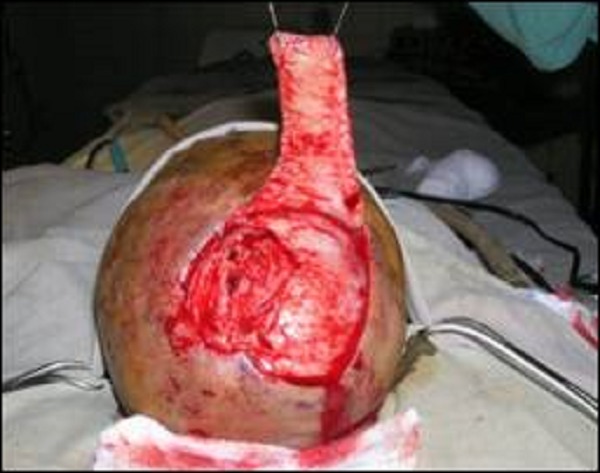
Figure 3: Flap elevated

**Figure:**
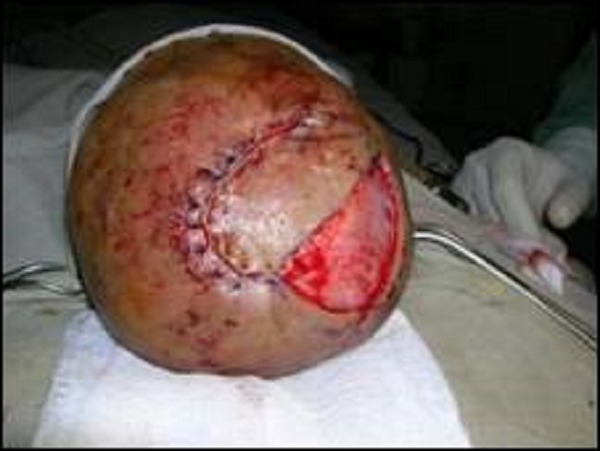
Figure 4: Flap inset

**Figure:**
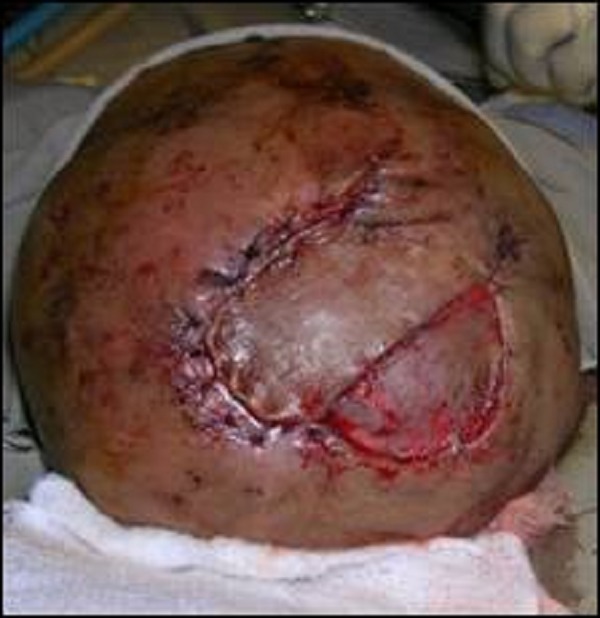
Figure 5: The donor site closure with split thickness skin graf

## DISCUSSION

ACC is a heterogeneous constellation of disorders that are characterized by the absence of a patch of skin over any part of body since birth. In 70% of cases it is a solitary lesion located on the vertex, a little lateral to the midline; however multiple patches of ACC are also documented in literature. It may involve about 0.5cm to 10 cm of the skin surface. The lesions may be rounded, elliptical, or stellate in configuration. Frieden classified these patients into 9 groups. Most of the patients belong to the Group 1 of the classification which is characterized by the scalp lesion in absence of associated multiple anomalies. Our patient was too belonged to the Group 1 based on the presence of isolated ACC over the scalp with no other abnormalities. Most of the patients with ACC present at birth with a lesion devoid of hairs and with a scarred or parchment like membrane extended throughout its base. Sometimes a collar of hair surrounds it – the hair collar sign. The other presentation is with an ulcer of variable depth over the scalp. In majority of cases only epidermis or dermis is involved; rarely the ulcer may involve underlying skull and meninges as happened in our case [1-4]. The lesion in our case was rounded and on the vertex involving the scalp, part of skull and meninges. By chance, the superior sagittal sinus was eroded and life threatening hemorrhage started.

The exact etiology is still unrevealed however it is considered as multifactorial. Genetic factors, environmental and teratogens are also considered in the etiology along with vascular compromise. Vijayashankar reported cases of ACC in a mother and son representing a hereditary process involved in the etiology [1,4].

Patients of ACC that involve merely epidermis or dermis usually born with a completely or partially healed lesion. Sometimes a small ulcerated area is the presentation. In these cases non-operative management is sufficient which is in the form of care of wound, wound cleanliness, and application of skin ointments like silver sulfadiazine etc. Rarely, surgical interventions have to be performed. The main indications for surgical interventions at birth are extensive lesion involving bone and meninges [1-4]. In our case the life threatening hemorrhage was the main indication for surgery. The superior sagittal sinus was repaired followed by covering with the transposition flap.

In short, ACC is a very rare disorder, rarer still is its presentation with life threatening hemorrhage. Early surgical intervention is recommended in cases where the lesion was involving the deeper structures as in our case.

## Footnotes

**Source of Support:** Nil

**Conflict of Interest:** None declared

